# Filling Children's Teeth

**Published:** 1894-12

**Authors:** W. G. A. Bonwill


ARTICLE VII.

### FILLING CHILDREN'S TEETH.

BY DR. W. G. A. BONWILL.

I never use gold in the temporary teeth, seldom amalgam or tin; save on grinding surfaces, when cavities are very small or very large and no pulp involved in the part, and oxiphosphate very seldom, and only where I can keep it perfectly dry. Not that any of these articles are not valuable, but the preparation of cavities and the situation of decay, the near approach to the pulp of nearly every proximal decay, and the age of the subject preclude their use. Never demoralize any youthful client by much excavation or formidable show of instruments, or by slow, sluggish movements. My aim is expedition; as few minutes in the chair as possible; inflicting but little pain and inconvenience,—gentleness, kindness, and yet positiveness.

My greatest ally as the filling-material is pink gutta-percha, such as is used for base-plate. Aside from its use for a stopping on all proximal surfaces, there is one grand object in view to be ever held in mind, the importance of the position of the first permanent molar when it emerges. Unless this base column, or abutment if you please, is not kept well back toward the ramus then irregularity will come to the incisors. It is not enough to merely stop decay and stuff in amalgam or oxiphosphate; we must keep the temporary molars from approaching each other more than normal, and prevent the alveolar processes from encroachment and absorption from direct pressure of the roots of the temporary molars, which is invariably the case when the proximating surfaces are cut by caries and allowed to trespass on each other. We cannot use a separator here to gain space, we dare not shape the cavities for a metal filling for fear of the pulp. What is to be done?

If possible, as soon as the least decay is noticed on the proximal surfaces and you can get in from the crown or on the buccal sides with the least excavating, by hand or machine, if it must be used, whether you can keep the cavity dry or allow it to remain moist, stuff in the gutta-percha forcibly between the teeth, smooth, and let alone to watch every three or six months. Where the cavities are large when you first see them, remove no decay over the pulp. Break down all superfluous walls, saturate with carbolic acid or creasote, force in a lump of the gutta-percha by filling all space as one filling, and let it go till the teeth have become so far separated by the act of mastication—not by expansion of the material—as to have replaced with another or a patch on the surface. Now, here is the point I wish to make that you have never recognized as a factor, because you have ignored the laws of articulation.

By this means I save from future decay and the risk of pulp exposure; but above all else I give a condition that enables the child to use with impunity every part of the jaws with hard or soft food, and no pain or fear of it, which no other plan could offer. And, above all this, I drive the first permanent molar so much farther back on the ramus that, the nearer it is to the condyle or point of motion, the wider it keeps the jaws apart at the incisors, and prevents the too great encroachment of the lower on the palatal surfaces of the upper incisors, which, if allowed, would destroy normal articulation,—make too deep an overbite and underbite, and, withal, cause an overlapping of the inferior incisors and the full use of the jaw teeth, because, in the lateral movements of the lower jaw, the incisors would strike first too long before the molars could come in contract, and really, only the up-and-down movement could be attached.

You will admit that recurrence of decay at the cervical border gives you the greatest difficulty to surmount, and as yet you have not reached the cause nor the remedy. If this one thing alone can be mastered, we have overcome our most powerful foe.

It is a fact not to be denied that every dentist cries out for some method to prevent recurrence at the cervix. This is positive proof that every one has his hands full of proximal cavities from the cuspid back. Every one must admit that contour fillings alone have been the only help or partial cure, though it has to be repeated or requires oft patching.

A case presents where caries have run wild. Not a proximal surface but is involved. No pulps quite exposed, but threatening. Every tooth has been filled and re-filled, and by more than one dentist. Contour has been attempted. Where the fillings of gold remain they are so undermind there is nothing but utter annihilation unless all are removed. The teeth, from their loss of proximate surfaces, are all out of articulation, which can be best seen by taking an impression and putting the casts in my articulator. Look closely at the cervix, and you will find the root of each so close that no thread can be forced through, and the decay is far up under the cervix. Look further, and probe for the alveolar process, and not a vestige of it remains for a quarter of an inch up. Look also to the second molar where the first has been extracted. On that side the process is gone far down and nothing but loose gum tissue remains, and is constantly receding, and wherever a tooth has been lost the process about the cervix absorbs as the body of the jaw absorbs.

In this state of affairs you put on your dam and separator, and you obtain a slight widening and at once fill permanently the excavated tooth. No attention is paid to the articulation of the teeth. You have no desire to wait, and you rush on headlong to fill and get your pay. The rest of the teeth are left without anything in them till one by one you have had your patient at least twice a week for months, two hours or more at a sitting, till they are exhausted and condemn dentistry, and while you are rushing through to complete every cavity with a filling you have done nothing to prevent further rapid decay, and pulps become exposed and the patient has to suffer.

Now, I know this is the case with nearly every man's practice. I see it every week, and I know from personal contact in conversations with patients of others who have not come to me for treatment.

Now, you can do better than this, and not only retain your patients but bridge over time as well as space, and fill and treat at your leisure.

I will take the same mouth just illustrated, and without placing in one single permanent filling of any kind of metal, treat it with pink gutta-percha alone, with a little of the white as a facing, where necessary. I cut out only partially the cavities on one side of the jaw or jaws, always exposing every grinding surface where the proximal is gone, and make compound by running all of the cavities into one, seldom leaving any proximal cavity to stand alone, but opening it into the grinding surfaces. This is a cardinal principle with me. There is one surface or border I complete at once, and that is the cervical, so that I never have to touch it again, and this I cut so far up as to not only remove all caries, but where I know the gum and process will grow up and over it. This is finished, and to enable me to do so I forgot to say I never put on the rubber-dam in any case till, I fill permanently, when the cervix is firm and will admit of its adjustment. It is easy to stop the blood with perchlorid of iron, creasote, or any styptic.

And now for further treatment. Into all the space I have made on one or more sides in one or more teeth I place great pieces of pink gutta percha, and with no separation between them in any case, stuff the whole intervening space, trim, and let alone. This I do till every place is filled in. I dismiss the patient and have him call in three or six months or a year, as I may please; and, as I find the teeth wide enough apart for a plus-contour filling, and the alveolar gum border and process is in perfect health, and the process has grown up to the gutta-percha, then I fill only those that show that they are far enough apart at the cervix to permit a healthy, full process to grow, in order that the gum will have proper substance, and cleave to the root and cover up and over the margin of the filling at the cervix. In this, I tell you, is your future security at the alveolar border.

No one has ever called attention to the difference in width of the proximal spaces at the cervix for the bicuspids and molars. The gutta-percha should remain in till double the width or space is gained between the molars than the bicuspids, on account of the greater size of the molars, where more proximal surface is in contact and no room left for cleansing, unless the spaces are very much greater than normal, and the contour made to suit this issue. Here is where you will say, "You will destroy the articulation and cause greater strain on the fillings and also the teeth." No; you are mistaken. When the whole of these proximal surfaces are filled with the semi-elastic stopping, and the act of mastication set up, the teeth that at first are out of the normal position and only touch on part of their crown surfaces are now allowed to readjust themselves; as the gutta-percha will give where the greatest pressure is brought to bear, and where least resistance is offered, no change occurs. I am not mistaken in this. Try it, and watch a few cases if you cannot believe me.

This method is a test for any further treatment, which, if needed, can so easily be done. It permits of weeks, months, and years before the permanent filling need be introduced. No danger of decay, none of loss of structure from fracture. And, in fact, you can dismiss the patients thus treated with the greatest indifference as to the issue. Do you ask whether I charge for all this work, and when I send in my bill? I charge for even my thoughts as well as my work. My patients never object, but often beg me to leave in the gutta-percha.

Thus I practice with all; and I am happy in this, knowing that I do far more good, am not troubled about immediate root filling,—fillings falling out,—"conservative treatment of dental pulp." Nor does pyorrhea ever invade on my domain of original work, because I know the value of articulation, and how to make every tooth perform its individual and collective function, and no undue pressure given it to press or work its life out of it and give rise to the denudation of the peridental membrane; nor is the food ever found pressing up into the cervical border and remaining, nor the cervix so weakened by want of contact with firm alveolar processes, and the gum is left to hug the root at this vital portion so tightly that nothing ever creeps in to cause recurrence at the cervix.

Any dentist who allows his original patient who follows orders to have pyorrhea should be sued for damages. See that no food presses on the gum border; see that no tooth is unduly pressed and contorted by false articulation, caused by improper width and contouring; allow no biting of threads, cracking of nuts, biting of ice on one tooth only; or, when a tooth, or teeth, has been lost, see that the articulation is restored,—and my word for it, gout or no gout, syphilis or disease, pyorrhea will not come, except filth and malaise of one or more teeth.

Gutta-percha used as a matrix for gold, amalgam, or oxiphosphate fillings I will not dwell on; you need nothing better. For holding teeth in position after correction where there are cavities in both, I need only mention it. As for assistance on the temporary and permanent teeth, to keep the ligature from slipping down on the cervix by carrying the ligature through it; for fastening pins into roots for crowns; as a medium between crowns and roots to prevent further caries; as a protection to all roots when a gold crown is used; and, in fact, as a factor in our practice, there is nothing to fill its place.

In only one instance it will not do. Never place it in contact with an amalgam filling, especially when it is covered up over a nearly exposed pulp, for it will oxidize the amalgam and discolor it and make it valueless at the margin. While gutta-percha is not so. The sulfur in the pink will do the work, hence I say this contact with amalgam is a failure. I am in love with it, and without it I would be lost.—*International Dental Journal.*

